# Navigating carbon neutrality: policy pathways and consistency on industrial decarbonization in China

**DOI:** 10.1186/s13021-025-00356-7

**Published:** 2025-12-14

**Authors:** Cheng Zhou, Wanhao Zhang, Clare Richardson-Barlow, Zhenhua Zhang

**Affiliations:** 1 School of Public Administration, Huaiyin Normal University, Huaian, China; 2 School of Government, Nanjing University, Nanjing, China; 3 Department of Interdisciplinary Studies of Culture, Norwegian University of Science and Technology, Trondheim, Norway; 4 School of Politics & International Studies, University of Leeds, Leeds, UK; 5 School of Economics, Lanzhou University, Lanzhou, China

**Keywords:** Climate change, Carbon neutrality, Environmental governance, Industrial decarbonization, Policy pathway, PMC

## Abstract

Climate change has intensified global demands for industrial decarbonization and carbon neutrality. As the world’s largest carbon emitter, China’s policy approach is pivotal to international climate governance and the low-carbon transition. This study conducts the first systematic evaluation of China’s industrial decarbonization policy framework established toward the carbon neutrality goal. Through a mixed-methods approach combining bibliometric analysis and Policy Modeling Consistency (PMC) Index, we analyze 58 national policy documents comprising approximately 610,000 Chinese characters. Results across five key decarbonization pathways show notable disparities in policy consistency: carbon emission abatement achieves perfect consistency (PMC-Index = 9.07), reflecting China’s prioritization of greenhouse gas emission controls, while energy efficiency (8.14) and scientific and technological innovation (8.12) demonstrate good consistency. By contrast, socio-economic risk mitigation (6.97) and circular economy (6.77) pathways only reach acceptable levels, revealing gaps in integrating just transition principles and industrial symbiosis. The asymmetric consistency stems from a misalignment across the five policy pathways, particularly the underdeveloped linkages between decarbonization, circularity, and socio-economic consideration. We recommend strengthening circular economy institutions through sector-specific material flow governance and industrial symbiosis networks, alongside proactive just transition policies such as skill development initiatives and compensatory mechanisms for vulnerable communities. This study contributes to theories of environmental governance and policy mixes, while offering globally applicable insights for reconciling emission reduction with industrial competitiveness and social equity.

**Supplementary Information:**

The online version contains supplementary material available at 10.1186/s13021-025-00356-7.

## Introduction

Climate change has emerged as one of the most critical challenges to human survival and public health in the twenty-first century [1, 2]. The Intergovernmental Panel on Climate Change (IPCC) has issued a stark “Code Red” warning, underscoring the urgent need for immediate decarbonization action at both global and national levels [3]. In response to this existential threat, the United Nations 2030 Agenda for Sustainable Development has positioned climate action as a foundational pillar, with SDG 13 specifically mandating the implementation of immediate, evidence-based interventions to mitigate climate warming and enhance societal resilience to its cascading effects [4]. International consensus now holds that achieving carbon neutrality is paramount for fulfilling SDG 13 [5, 6]. As the world’s largest carbon emitter, China has committed to achieving carbon neutrality by 2060, a pledge that carries significant implications for global climate sustainability [7, 8]. To operationalize this goal, China has instituted a comprehensive policy framework to navigate its ongoing carbon neutrality initiatives [9].

Historical evidence suggests that industrial decarbonization, while environmentally necessary, may precipitate socioeconomic risks such as inequities and systemic vulnerabilities [10, 11]. For instance, the closure of carbon-intensive industries, such as fossil fuel extraction, has frequently led to adverse consequences, including energy poverty, reduced tax revenues, and, most acutely, large-scale unemployment. A salient example is Colombia’s La Guajira coal mines, where rapid decarbonization has rendered thousands of workers and their communities vulnerable to becoming “ecological refugees” [12]. Consequently, it has become critical to mitigate these risks by ensuring a just transition that aligns with national development priorities and generates decent work opportunities [13, 14]. Scholars increasingly emphasize that integrating just transition principles into industrial decarbonization policies is essential to addressing these socioeconomic challenges effectively [14, 15].

Chinese policymakers have likewise recognized the potential risks accompanying industrial decarbonization. In its pursuit of the carbon neutrality goal, China has introduced a more robust and holistic policy framework designed to preempt major socioeconomic disruptions. This evolving landscape signals a new phase in China’s industrial decarbonization trajectory. Yet, despite its significance, scholarly inquiry has not systematically examined industrial decarbonization through the lens of carbon neutrality policies. Prior research suggests that policy analysis can elucidate the role of governmental authorities in facilitating (or hindering) decarbonization efforts [16, 17]. Moreover, policy text analysis has proven instrumental in evaluating policy quality and the feasibility of stated objectives [18]. Globally, the efficacy of industrial decarbonization hinges on sound policymaking, with robust policies demonstrably reducing emissions, while poorly designed ones can undermine progress toward carbon neutrality [19, 20].

To address this gap, this research employs a mixed-methods approach that integrates bibliometric analysis with the PMC-Index. This methodology enables a systematic evaluation of the policy pathways and consistency within China’s industrial decarbonization and carbon neutrality framework. Within these policy pathways, policy consistency emerges as a critical metric for evaluating their effectiveness, serving as a foundational indicator of the policy framework’s internal coherence and implementation robustness. Successful complex, long-term transitions like industrial decarbonization require a consistent policy design to align instruments across governance dimensions. This alignment is fundamental, as it provides clear signals to stakeholders, enables predictable enforcement, and ultimately ensures the achievement of core policy goals. This focus is particularly salient given China’s dual role as the world’s leading emitter and a proactive architect of carbon neutrality policies [20, 21]. By systematically dissecting the policy landscape, this study offers critical insights into the challenges and opportunities in implementing industrial decarbonization efforts, with broader implications for other developing nations. To anchor this investigation, we formulate the following overarching research question: What are the policy pathways and levels of policy consistency within China’s industrial decarbonization framework as it pursues its 2060 carbon neutrality goal? This central question is operationalized through three specific inquiries: (1) What distinct policy pathways constitute China’s strategy for industrial decarbonization? (2) How consistent are policies within each of these identified pathways? (3) What governance strengths and critical gaps are revealed by disparities in policy consistency across different pathways? These questions guide our inquiry and frame the study’s three key contributions to the relevant field:

Theoretically, this study contributes to environmental governance and policy mix design theory by systematically evaluating policy consistency across China’s five industrial decarbonization pathways, revealing its distinctive “differentiated governance logic”. Existing research has predominantly treated policy consistency as monolithic or focused on advanced economies, neglecting the asymmetric consistency patterns in state-led transitions. By applying pathway-specific PMC-Index analysis, we demonstrate how China’s hybrid regulatory-market approach achieves perfect consistency in emission abatement but lags in circular economy integration, thereby extending the theoretical foundations of both environmental governance and policy mix design.

Methodologically, we introduce a novel mixed-methods framework combining bibliometric analysis of 610,000 + Chinese characters in policy texts with quantitative PMC-Index modeling. Prior studies often rely on single-tool approaches or aggregate policy evaluations, overlooking pathway-specific disparities. Our mixed-methods approach enables granular mapping of policy pathways while the multi-dimensional PMC-Index quantifies consistency gaps, thereby setting a benchmark for future policy analysis.

Practically, the study delivers actionable policy solutions by identifying and addressing critical implementation gaps in China’s industrial decarbonization framework. Existing research has largely overlooked the operational challenges of translating national environmental goals into effective local actions, particularly in integrating circular economy and just transition principles. Through systematic analysis of policy documents, we demonstrate how strengthening institutional coordination and developing place-based transition programs can enhance policy effectiveness while ensuring the just transition.

The rest of the paper is organized as follows. Sect. "Literature Review" offers a literature review on carbon neutrality and industrial decarbonization. Sect. "Data and Methods" describes policy data sources and methods. Sect. "Results" presents the key results, including the identified policy pathways and their consistency evaluations. Sect. "Discussion and Implications" discusses the implications of these results. Finally, in Sect. "Conclusions", we conclude the paper with key findings.

## Literature review

The pursuit of carbon neutrality has emerged as a dominant paradigm in climate change mitigation research, generating a substantial and rapidly evolving body of literature across multiple disciplines [5, 22]. As growing numbers of countries declare carbon neutrality targets, academic inquiry has expanded from initial conceptual explorations to sophisticated analyses of implementation pathways and their systemic implications [23, 24]. This scholarly discourse has become increasingly nuanced, recognizing carbon neutrality not merely as a technical challenge but as a complex socio-technical transition requiring coordinated action across technological, socioeconomic, and governance dimensions: (1) Technological pathway research has evolved from single-technology assessments to systemic decarbonization studies, spanning renewable integration [25], CCUS scale-up challenges [26], and industrial electrification [27]. (2) Socioeconomic studies now examine transition justice through energy poverty [14], regional disparities [28], and labor market impacts [29], developing comprehensive justice frameworks [30]. (3) Governance research compares policy instruments [31] and analyzes decarbonization pathways [23], emphasizing policy synergy [32], but often lacks a focus on carbon neutrality policy text analysis [20].

Industrial decarbonization has emerged as a critical frontier in carbon neutrality research, given the sector’s substantial emissions and the technical complexities of abatement [33]. Current scholarship primarily adopts two complementary analytical lenses. The first stream conducts granular sectoral analyses, particularly of hard-to-abate industries where emission reductions are most challenging. Seminal works not only examine breakthrough technologies in steel production [34], alternative binders in cement manufacturing [35], and feedstock transitions in chemical industries [36], but also identify key barriers such as capital inertia and supply chain dependencies. The second stream investigates systemic transition frameworks that enable cross-sectoral decarbonization. This includes research on circular economy models that optimize material flows [37], institutional designs for just transitions that protect vulnerable workers [13], and industrial symbiosis networks that facilitate resource sharing [38]. Recent work has begun integrating these approaches through socio-technical transition frameworks.

Despite these advancements, nevertheless, significant gaps persist. First, the extant literature shows a pronounced bias toward advanced economies in studies of industrial transitions, largely neglecting the state-led approaches prevalent in developing nations like China. This neglect is especially problematic considering these countries’ increasing dominance in global industrial output. Second, existing studies disproportionately focus on technological or economic dimensions while failing to provide a systematic analysis of policy pathways and consistency. This gap specifically concerns how decarbonization policies evolve and interact within national environmental governance frameworks. Understanding policy pathways is essential for tracing the evolution and sequencing of environmental governmental interventions, which collectively shape long-term sustainable transition trajectories [20]. Building on this, policy consistency serves as a crucial theoretical and technical tool for analyzing the internal coherence of policies across multiple pathways, identifying their strengths and weaknesses to address societal challenges across different contexts [39]. Evaluating policy consistency is vital as it determines the alignment between individual policy instruments and overarching strategic goals, thereby influencing the feasibility and effectiveness of complex socio-technical transitions like industrial decarbonization. Third, China’s industrial decarbonization policies in its latest carbon neutrality phase remain underexplored, despite their global significance given the nation’s dual status as the world’s manufacturing hub and largest emitter.

This study bridges these gaps by employing a novel mixed-methods approach: bibliometric analysis to map China’s policy pathways, complemented by PMC-Index modeling to quantify policy consistency in industrial decarbonization toward the 2060 carbon neutrality goal. By measuring policy variables such as policy nature, instruments, incentives, and support, the PMC-Index provides a quantifiable measure of how well policy designs incorporate multi-dimensional governance. Such an approach not only advances methodological innovation in policy studies but also yields practical insights for developing economies navigating the trilemma of industrial competitiveness, emission reduction, and social equity.

## Data and methods

### Data

**Fig. 1:**
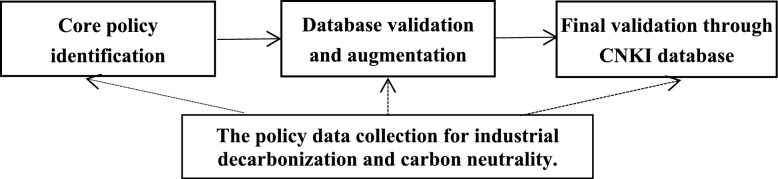
The data collection process

This study employs a tripartite data collection methodology to achieve comprehensive coverage of China’s policy framework for industrial decarbonization toward carbon neutrality. The analysis focuses on the policy period from November 2012 to December 2024. This timeframe begins with the 18th National Congress of the Communist Party of China in November 2012, a historic turning point that marked the official integration of “ecological civilization construction” into the national development strategy, initiating a systemic shift in China’s environmental governance and decarbonization policy framework. The period concludes in December 2024, by which time the foundational policy framework for navigating carbon neutrality had been substantially established at the national level. We systematically screened 58 pivotal policy documents issued during this period, which collectively form the nation’s industrial decarbonization and carbon neutrality framework (Additional file 1). This process resulted in a corpus spanning 610,000 Chinese characters for analysis. Our rigorous data acquisition protocol, illustrated in Fig. 1, involved three methodologically distinct stages:

1. Core policy identification. We conducted exhaustive searches of Chinese national agencies’ official portals, employing a carefully constructed keyword taxonomy encompassing: decarbonization, industrial transition, carbon peaking, carbon neutrality, net-zero emissions, carbon mitigation, climate change, low-carbon development, and ecological civilization. This initial collection established our baseline policy corpus.
2. Database validation and augmentation. We subsequently cross-referenced and supplemented our initial findings through searches of official government policy databases. These standardized repositories of national-level policy documents enabled comparative analyses to verify the accuracy and enhance comprehensiveness. This phase served as a critical quality control measure, ensuring that no significant policies were overlooked.
3. Final validation through CNKI database. To confirm data comprehensiveness and representativeness, we conducted supplementary searches using the China National Knowledge Infrastructure (CNKI), the nation’s most extensive and authoritative academic database [15]. This final verification stage ensured that our collection fully encompasses the current landscape of Chinese carbon neutrality policies while maintaining strict relevance to industrial decarbonization objectives.

This multi-method approach achieves three critical objectives: (1) comprehensive policy documentation through source triangulation, (2) enhanced data validity via cross-verification mechanisms, and (3) establishment of methodological standards for policy ecosystem analysis. Our approach complements recent efforts to systematically document China’s low-carbon policy intensity across multiple governance levels [40]. By mitigating the inherent biases of single-source studies, the protocol offers both immediate empirical robustness and long-term value as a replicable research framework for comparative policy studies [41]. Notably, to maintain analytical rigor and focus, we adopted a selective extraction protocol for broad-scope documents. For instance, from the “Report of the 20th National Congress of the Communist Party of China”, we specifically extracted only those sections directly pertinent to our research focus—notably the segment addressing “Promoting green development and harmonious coexistence between humanity and nature”.

### Methods

This research employs an integrative mixed-methods [42] design to comprehensively analyze China’s carbon neutrality policy framework. Our methodology synthesizes bibliometric analysis for policy pathways mapping with quantitative PMC-Index evaluation, capitalizing on their synergistic advantages to strengthen analytical validity and interpretive depth.

1. **Bibliometric analysis: Clarifying policy pathways of industrial decarbonization toward carbon neutrality**

This study utilizes bibliometric analysis to systematically examine the policy pathways of industrial decarbonization within China’s carbon neutrality framework. Our methodological approach combines two complementary analytical tools—ROST CM6.0 for Chinese-language text processing and VOSviewer 1.6.19 for network visualization—to provide comprehensive insights into China’s decarbonization policy architecture.

First, this study employed a rigorous bibliometric analysis methodology utilizing ROST, a specialized Chinese-language text mining platform renowned for its superior preprocessing capabilities in handling complex Mandarin policy documents [24]. The analytical process began with the systematic conversion of 58 policy documents (totaling 610,000 + Chinese characters) from their native formats (e.g.,.pdf,.doc,.html) into standardized text files, followed by comprehensive data cleaning procedures that included synonym normalization and the removal of non-substantive linguistic elements such as function words, ordinal indicators, and other grammatical particles.

Second, the refined textual corpus was subsequently converted into RefWorks-formatted documents for advanced network analysis in VOSviewer, selected for its demonstrated superiority in policy network visualization compared to alternative platforms like CiteSpace or COOC [43]. VOSviewer’s analytical framework employed three validated bibliometric measures: occurrence, links, and total link strength, enabling precise mapping of the semantic architecture underlying China’s industrial decarbonization policies.

This dual-platform bibliometric analysis represents a significant methodological advancement over conventional single-tool approaches, combining ROST’s unparalleled Chinese character recognition with VOSviewer’s sophisticated network analysis algorithms to overcome the unique challenges of Mandarin policy text analysis while ensuring rigorous, replicable results [15].

2) **PMC Index of industrial decarbonization**

This study advances the bibliometrically-identified policy pathways by applying the PMC Index methodology to quantitatively evaluate policy consistency across China’s five major industrial decarbonization trajectories. The PMC framework’s multidimensional quantification approach enables systematic assessment of policy characteristics, offering a validated mechanism for consistency measurement in complex transition contexts [39]. Our application of this methodology, with its pathway-specific analytical design, represents a significant advancement over conventional approaches [41].

We conducted the PMC evaluation in three methodologically rigorous stages [44–46].

In the first stage, building on established PMC literature and informed by policy studies [45, 46], we constructed an evaluation matrix comprising 10 primary and 48 secondary policy variables (Table 1). Our rationale for employing this detailed classification stems from the unique characteristics of Chinese policy documents, which often employ a rich and nuanced vocabulary to convey distinct policy intents and functional orientations [47]. For instance, “description” denotes the objective presentation of facts or situations, “supervision” implies monitoring and oversight functions, “guidance” offers directional advice without strict mandates, while “refinement” suggests improving the quality, precision, or effectiveness of existing mechanisms. This nuanced classification, consistent with methodological approaches in Chinese policy analysis, allows us to more accurately capture the subtleties in policy design and intent [41, 47]. Policy nature (X_1_) thus assesses the strategic intent and functional orientation of a policy, categorized by these specific functions [39, 46].

**Table 1:** The identification of policy variables

Main-variable	Sub-variable
X_1_: Policy nature	X_1:1_: Description; X_1:2_: Supervision; X_1:3_: Guidance; X_1:4_: Suggestion; X_1:5_: Encouragement; X_1:6_: Specification; X_1:7_: Optimization; X_1:8_: Enhancement; X_1:9_: Refinement
X_2_: Policy timeliness	X_2:1_: Long-term; X_2:2_: Mid-term; X_2:3_: Short-term; X_2:4_: Temporary
X_3_: Policy release agency	X_3:1_: CCCPC; X_3:2_: SC; X_3:3_: NDRC; X_3:4_: MEE; X_3:5_: MIIT; X_3:6_: SAMR; X_3:7_: Other departments
X_4_: Policy implementation agency	X_4:1_: State-level ministries and commissions; X_4:2_: Provincial governments; X_4:3_: Municipal governments; X_4:4_: County governments; X_4:5_: Township governments; X_4:6_: Village committees
X_5_: Policy incentive	X_5:1_: Financial subsidies; X_5:2_: Investment stimulus; X_5:3_: Tax reduction and exemption; X_5:4_: Loan allowance
X_6_: Policy instrument	X_6:1_: Command-and-control instruments; X_6:2_: Market-based instruments; X_6:3_: Voluntary instruments
X_7_: Policy support	X_7:1_: Science and technology support; X_7:2_: Information support; X_7:3_: Infrastructure construction; X_7:4_: Financial inputs; X_7:5_: Education and training; X_7:6_: Pilot demonstration and application
X_8_: Policy area	X_8:1_: Economy; X_8:2_: Society; X_8:3_: Environment; X_8:4_: Politics; X_8:5_: Technology
X_9_: Policy object	X_9:1_: Local government; X_9:2_: Enterprise; X_9:3_: Social organization; X_9:4_: Public
X_10_: Policy accessibility	Without any sub-variable

Policy timeliness (X_2_) evaluates the policy’s operational timeframe, distinguishing between long-term (over 5 years), mid-term (3–5 years), short-term (1–3 years), and temporary (less than 1 year) horizons. Policy release agency (X_3_) identifies the authoritative source of the policy [48], which includes key national bodies such as the Central Committee of the Communist Party of China (CCCPC), the State Council (SC), the National Development and Reform Commission (NDRC), the Ministry of Ecology and Environment (MEE), the Ministry of Industry and Information Technology (MIIT), the State Administration for Market Regulation (SAMR), and other departments [46]. Policy implementation agency (X_4_) specifies the level of government tasked with executing the policy, ranging from state-level ministries down to provincial, municipal, county, township governments, and even village committees [41]. Policy incentive (X_5_) examines the presence of economic instruments designed to spur compliance and adoption, including financial subsidies, investment stimulus, tax reductions and exemptions, and loan allowances [49].

Policy instrument (X_6_) classifies the primary governance approach employed, including command-and-control regulation, market-based mechanisms, or voluntary instruments [20, 50]. Policy support (X_7_) determines the types of ancillary resources committed to facilitate implementation, encompassing science and technology support, information support, infrastructure construction, financial inputs, education and training, and pilot demonstrations [45]. Policy area (X_8_) maps the policy’s scope of impact across key domains: economy, society, environment, politics, and technology [51]. The sub-variable politics specifically identifies content related to top-down hierarchical control, bureaucratic accountability, and the reinforcement of Party-state leadership structures in guiding the transition, distinct from the broader political nature of the policy process itself [46, 52]. Policy object (X_9_) identifies the primary target groups addressed by the policy, such as local government, enterprises, social organizations, and the public [49]. Policy accessibility (X_10_) is a binary assessment of whether the policy document is publicly available, with no further sub-variables [46].

This dual-layered framework uniquely integrates universal policy assessment dimensions with distinctive characteristics of China’s governance system, overcoming the contextual blind spot prevalent in existing evaluation approaches.

Second, we operationalized the industrial decarbonization policy analysis through a analytical multi-input–output framework [39, 41, 51]. Each sub-variable was dichotomously coded, enabling systematic quantification of policy characteristics while maintaining analytical transparency.

Third, the PMC-index computation follows a four-stage protocol:

In Step A, binary coding is systematically applied to all policy sub-variables [49]. As expressed in Formula (1), $X\sim N\left[ {0,1} \right]$ indicates the binary assignment (1 = presence; 0 = absence) for each sub-variable. Formula (2), $XR:\left[ {0\sim 1} \right]$, defines the codomain of the normalized policy variable $XR$ as the continuous interval between 0 and 1 [41]. This formulation ensures that all sub-variables, after initial binary assignment, are transformed into normalized scores within the [0, 1] range. Such normalization facilitates consistent aggregation and comparability across the diverse policy variables used in the evaluation.

Step B involved main-variable quantification [46, 51]. Through Formula (3), primary variables were normalized to [0, 1] intervals. Here, $X_{i}$ denotes the *i*th main-variable and $X_{ij}$ represents its *j*th sub-variable, with *i* denoting the total number of main-variables and *j* indicating the ordinal position of a sub-variable within the main-variable. The function $T\left( {X_{ij} } \right)$ signifies the total number of sub-variables contained within the main-variable.

In Step C, the composite index was constructed [45, 53]. Formula (4) generated pathway-specific PMC-Indices (scale: 0–10), with the following classification thresholds: Low Consistency (0–4.99); Acceptable Consistency (5.00–6.99); Good Consistency (7.00–8.99); Perfect Consistency (9.00–10.00).

Step D: The PMC surface is graphically constructed according to Formula (5), which allows for the visualization of the policy consistency results within different industrial decarbonization pathways.

1 $$X\sim N\left[ {0,1} \right]$$

2 $$X = \left\{ {XR:\left[ {0\sim 1} \right]} \right\}$$

3 $$X_{i} \left[ {\mathop \sum \limits_{j = 1}^{n} \frac{{X_{ij} }}{{T\left( {X_{ij} } \right)}}} \right]$$

4 $$\begin{aligned} {\text{PMC - Index}} & = \mathop \sum \limits_{i = 1}^{m} \left( {X_{i} \left[ {\mathop \sum \limits_{j = 1}^{n} \frac{{X_{ij} }}{{T\left( {X_{ij} } \right)}}} \right]} \right) = \left[ {X_{1} \left( {\mathop \sum \limits_{j = 1}^{9} \frac{{X_{1j} }}{9}} \right)} \right. \\ & \quad + X_{2} \left( {\mathop \sum \limits_{j = 1}^{4} \frac{{X_{2j} }}{4}} \right) + X_{3} \left( {\mathop \sum \limits_{j = 1}^{7} \frac{{X_{3j} }}{7}} \right) \\& + X_{4} \left( {\mathop \sum \limits_{j = 1}^{6} \frac{{X_{4j} }}{6}} \right) \\ & \quad + X_{5} \left( {\mathop \sum \limits_{j = 1}^{4} \frac{{X_{5j} }}{4}} \right) + X_{6} \left( {\mathop \sum \limits_{j = 1}^{3} \frac{{X_{6j} }}{3}} \right) \\& + X_{7} \left( {\mathop \sum \limits_{j = 1}^{6} \frac{{X_{7j} }}{6}} \right) \\ & \quad \left. { + X_{8} \left( {\mathop \sum \limits_{j = 1}^{5} \frac{{X_{8j} }}{5}} \right) + X_{9} \left( {\mathop \sum \limits_{j = 1}^{4} \frac{{X_{9j} }}{4}} \right) + X_{10} } \right] \\ \end{aligned}$$

5 $$PMC - Surface = \left[ {\begin{array}{*{20}c} {X_{1} } & {X_{2} } & {X_{3} } \\ {X_{4} } & {X_{5} } & {X_{6} } \\ {X_{7} } & {X_{8} } & {X_{9} } \\ \end{array} } \right]$$

Our study contributes to policy evaluation methodology by implementing a multi-pathway PMC-Index framework, representing a substantive improvement beyond conventional analytical approaches [41]. Unlike previous studies employing singular PMC analyses on aggregated policy portfolios [45, 46, 54], our research implements five discrete evaluations aligned with specific decarbonization pathways. Though computationally intensive, this disaggregated framework provides superior explanatory power by revealing China’s distinctive “differentiated governance logic”—where policy instruments and implementation mechanisms vary systematically across decarbonization pathways. The approach not only enhances analytical precision but also provides critical insights into how China’s unique institutional context shapes industrial transition pathways, addressing a significant gap in current policy evaluation literature.

## Results

The results of the study encompass two main areas: (1) the identification of distinct industrial decarbonization pathways, and (2) the evaluation of policy consistency levels across these pathways.

### Bibliometric visualization of policy pathways within industrial decarbonization

**Fig. 2:**
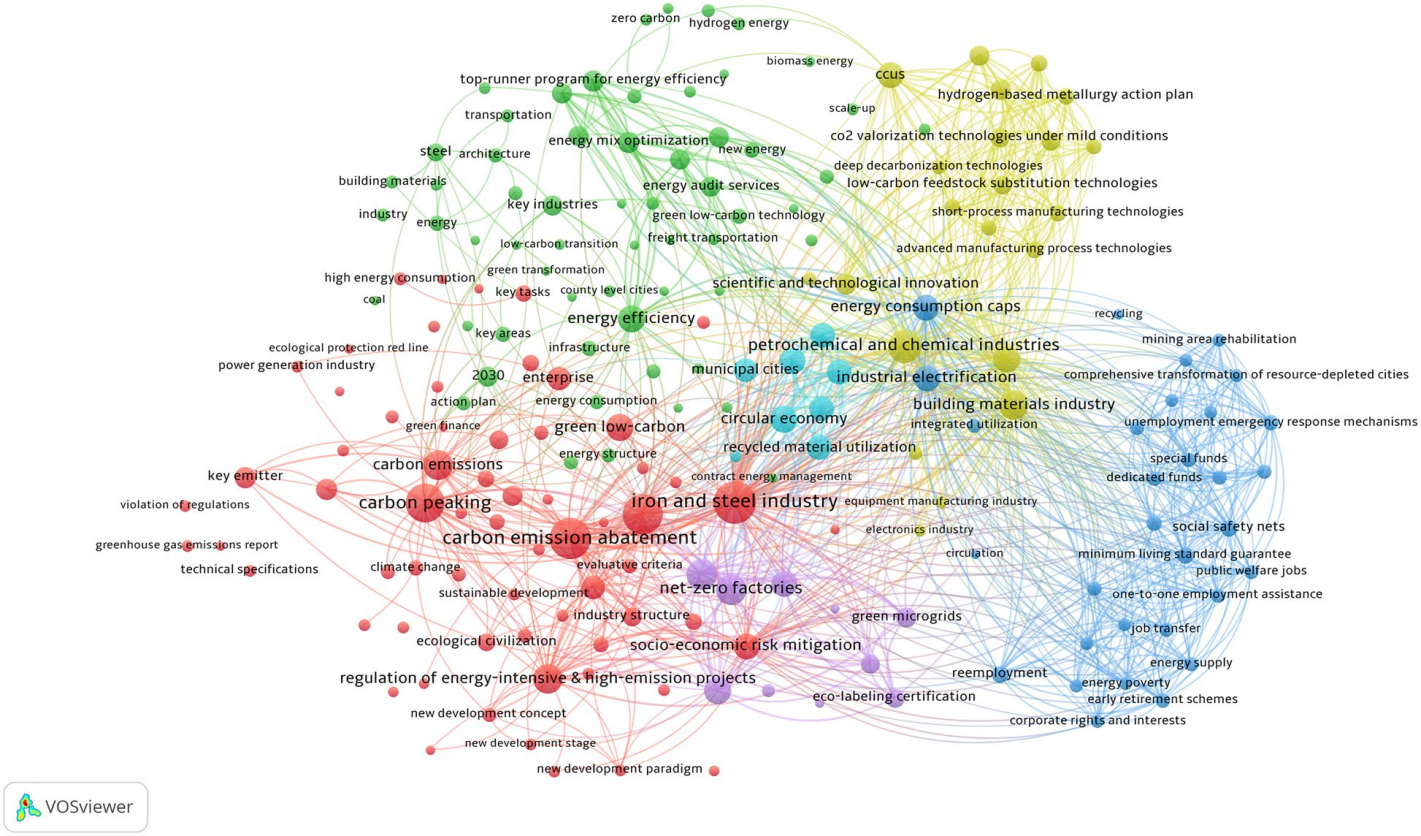
Bibliometric visualization of policy pathways within industrial decarbonization. The minimum number of keyword occurrences was set to 7. The nodes represent keywords, with size proportional to occurrence frequency. The line between any two nodes in this bibliometric visualization indicates the link between two keywords

After importing the cleaned data into VOSviewer, we identified 196 policy keywords pertaining to industrial decarbonization. The bibliometric visualization yielded six distinct clusters, with each cluster assigned a different color for representation (Fig. 2). The keyword composition within each cluster demonstrates strong internal correlation and thematic significance. As can be seen from the Fig. 2, five primary policy pathways for China’s industrial decarbonization emerge: energy efficiency (green cluster), carbon emission abatement (red cluster), circular economy (cyan cluster), scientific and technological innovation (yellow cluster), and socio-economic risk mitigation (blue cluster).

Energy efficiency constitutes a foundational element of China’s industrial decarbonization strategy. This pathway involves comprehensive optimization of the national energy mix, with a pronounced shift toward cleaner electricity sources and accelerated industrial electrification to displace fossil fuel consumption. Policy enforcement includes mandatory energy consumption caps, reinforced through systematic energy conservation oversight and auditing mechanisms applied to high energy-consuming sectors. Initiatives such as the Top-Runner Program establish sector-specific benchmarks to incentivize enterprises to adopt energy-efficient retrofits of key industrial equipment. Additionally, differentiated electricity pricing introduces market-based penalties for inefficiency, thereby aligning economic incentives with decarbonization objectives. Together, these measures aim to achieve a dual objective crucial for transitional economies: lowering energy intensity while boosting (or preserving) industrial competitiveness.

Carbon emission abatement is pursued through stringent regulatory constraints on energy-intensive and high-emission projects, coupled with targeted incentives for low-carbon industrial transformation. This approach is embodied in the expansion of net-zero factories and green industrial parks, which integrate renewable energy procurement and decentralized green microgrids to elevate the share of clean energy. Further progress in supply chain decarbonization is achieved through eco-labeling certifications that embed lifecycle carbon criteria into procurement standards. These measures reflect an emerging global paradigm favoring green industrial ecosystems, where regulatory and market-based mechanisms operate collaboratively to accelerate emission reductions.

The circular economy pathway seeks to decouple industrial output from primary resource extraction by promoting low-carbon material substitution and recycling industrial solid waste. Remanufacturing, particularly of mechanical and electrical products, is prioritized to extend product lifespans, while the utilization of recycled materials is scaled up via tax incentives and extended producer responsibility schemes. These strategies align with the concept of industrial symbiosis, by which waste streams are reconceptualized as inputs for neighboring processes, as a crucial mechanism for achieving circularity in heavy industries.

Scientific and technological innovation serves as a critical enabler of China’s decarbonization agenda, supported by strategic R&D investments in breakthrough areas such as hydrogen-based metallurgy and mild-condition CO_2_ reduction. Industrial process reengineering, such as the adoption of short-process manufacturing and advanced equipment retrofitting, reduces systemic energy demand. Meanwhile, digitalization technologies, including AI-driven energy management systems, optimize real-time operational efficiency. Concurrently, carbon capture, utilization, and storage (CCUS) and other deep decarbonization technologies target residual emissions in hard-to-abate sectors including petrochemicals, chemicals, iron, and steel. This comprehensive innovation strategy echoes scholarly advocacy for integrated “technology-policy bundles” to overcome industrial lock-in effects [35].

Socio-economic risk mitigation has been institutionalized to address transition-induced disruptions. Workforce transition programs, such as vocational training, job relocation support, and entrepreneurship incubation, aim to mitigate labor market disruptions in vulnerable regions, especially those reliant on coal. Social safety nets are strengthened through unemployment early-warning systems and emergency relief funds to protect vulnerable groups. Regional revitalization efforts, such as ecological remediation of mining zones and industrial diversification in resource-depleted cities, are deployed to mitigate spatial inequities. These measures resonate with the academic emphasis on just transition frameworks, in which policy alignment between decarbonization and social equity is essential for maintaining public trust and procedural legitimacy.

### PMC-Index results of policy consistency on industrial decarbonization

To account for the inherent heterogeneity in China’s industrial decarbonization policy logic and to enhance the scientific rigor of our analysis, we conducted five parallel PMC-Index evaluations corresponding to distinct policy pathways: (1) energy efficiency, (2) carbon emission abatement, (3) circular economy, (4) scientific and technological innovation, and (5) socio-economic risk mitigation (Table 2). This disaggregated analytical approach represents a significant methodological improvement over conventional single evaluations of policy portfolios [39], enabling more nuanced assessment of China’s decarbonization governance.

**Table 2:** The PMC-Index of five policy pathways within China’s industrial decarbonization

Item	Energy efficiency	Carbon emission abatement	Circular economy	Scientific and technological innovation	Socio-economic risk mitigation	Overall
Policy nature	0.81	0.86	0.43	0.81	0.57	0.70
Policy timeliness	0.78	0.92	0.39	0.73	0.59	0.68
Policy release agency	0.82	0.89	0.38	0.63	0.35	0.61
Policy implementation agency	0.68	0.93	0.71	0.81	0.72	0.77
Policy incentive	0.85	0.97	0.93	0.94	0.69	0.88
Policy instrument	0.79	0.89	0.69	0.77	0.68	0.76
Policy support	0.89	0.82	0.63	0.86	0.93	0.83
Policy area	0.79	0.96	0.78	0.75	0.75	0.81
Policy object	0.73	0.83	0.83	0.82	0.69	0.78
Policy accessibility	1.00	1.00	1.00	1.00	1.00	1.00
PMC-Index	8.14	9.07	6.77	8.12	6.97	7.81
Ranking	2	1	5	3	4	/

**Fig. 3:**
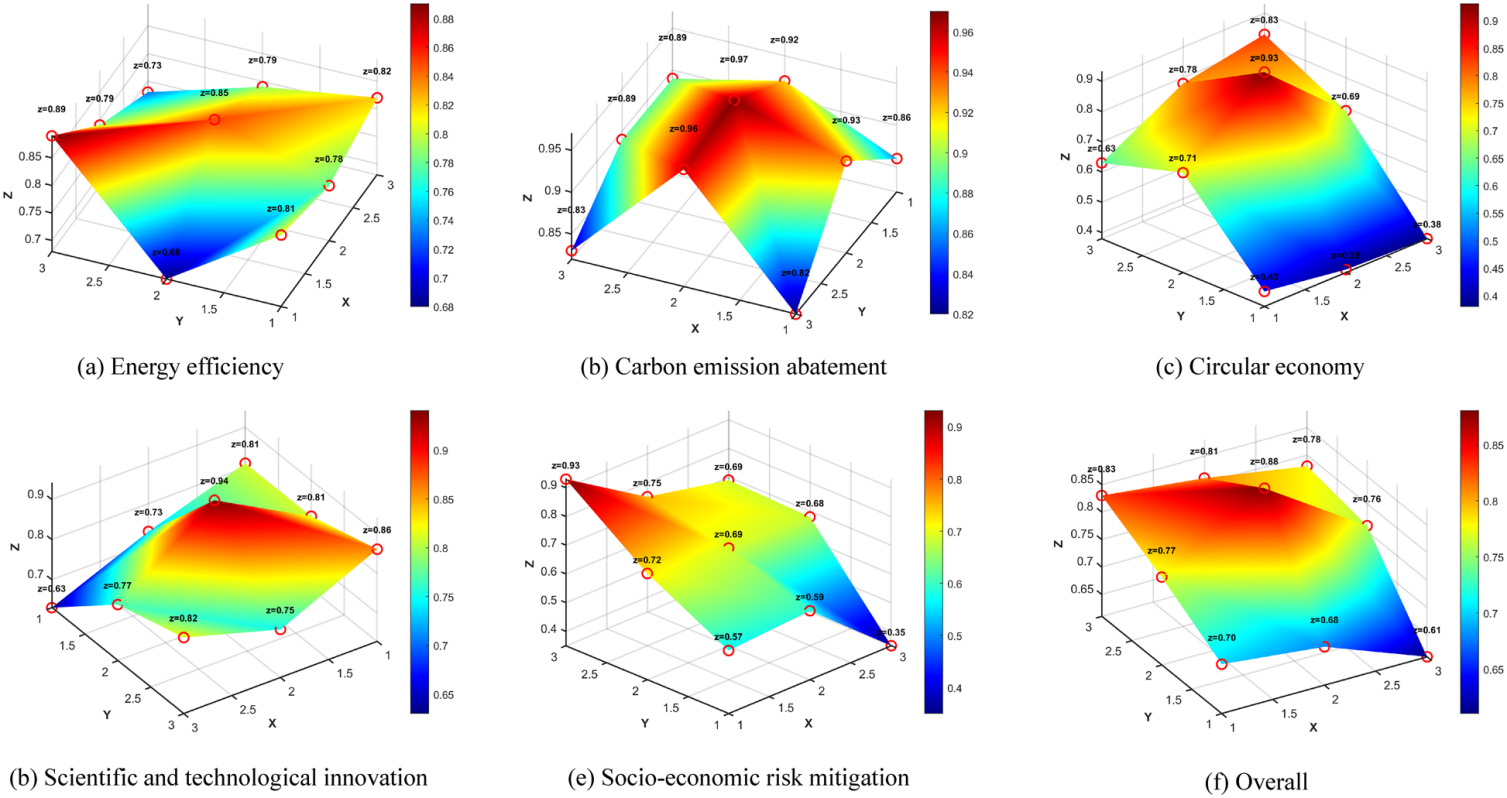
PMC-Surface of industrial decarbonization

The calculated PMC-Index scores, as systematically presented in Table 2, reveal noticeable variation in policy consistency across the five decarbonization pathways. The carbon emission abatement pathway achieved exceptional consistency (PMC-Index = 9.07), which is classified as Perfect Consistency (scores 9.00–10.00). This reflects China’s strategic prioritization of direct emission control measures through its hybrid regulatory-market approach. Both the energy efficiency (8.14) and scientific and technological innovation (8.12) pathways demonstrated Good Consistency (7.00–8.99), indicating robust but less comprehensive policy frameworks. In contrast, the socio-economic risk mitigation (6.97) and circular economy (6.77) pathways showed only Acceptable Consistency (5.00–6.99), suggesting relative weaknesses in integrating social equity and industrial ecology principles, respectively. Figure 3 provides a visual representation of these pathway-specific consistency profiles through PMC surfaces, offering an intuitive graphical summary of the disparities. As illustrated in Fig. 3, the X-axis represents the columns of the matrix (given in Formula 5 in Sect. "Method"), the Y-axis denotes the rows of the matrix, and the Z-axis indicates the normalized parameter values [0, 1] of the policy variables. In the PMC-Surface, distinct color blocks correspond to different numerical scores of the indicators: protruding areas reflect higher scores for the corresponding policy variables, while depressions indicate relatively lower scores. This visualization not only enhances the interpretability of the PMC-Index results but also facilitates comparative analysis across policy dimensions.

A granular analysis at the variable level yields critical insights into the architecture of China’s decarbonization governance model. The universal maximum score (1.00) for policy accessibility across all pathways confirms the public availability of policy documents, and aligns with prevailing administrative transparency norms. More notably, policy incentive scores (0.88) ranked highest among the substantive variables, indicating a strategic shift from pure command-and-control regulation toward incentive-based governance—a transition that aligns with global best practices for industrial decarbonization. This shift is manifested through diverse instruments, including tax incentives for energy-efficient technologies, green credit mechanisms for low-carbon projects, and tiered electricity pricing systems.

Conversely, policy release agency scores (0.61) emerged as the weakest substantive variable, exposing institutional coordination and cross-sectoral collaboration challenges. The policy textual analysis reveals an increase in multi-agency joint issuance, including instances of NDRC-MEE collaborations with other ministries. Despite this trend, policy formulation remains dominated by macro-regulatory agencies, particularly the NDRC and MEE, rather than sector-specialized bodies. The limited involvement of the MIIT in leading carbon neutrality policies is particularly concerning. This oversight is notable even though MIIT holds primary responsibility for industrial transformation and possesses critical technical expertise. This institutional imbalance risks creating critical policy-implementation gaps, as evidenced by slower progress in hard-to-abate sectors like manufacturing and fossil industries where MIIT’s operational knowledge is most crucial.

The results collectively underscore the complexity and multifaceted nature of China’s industrial decarbonization policies. The PMC-Index analysis reveals a clear strategic emphasis on emission control, with comparatively less comprehensive and consistent frameworks for energy efficiency and technological innovation. The lower consistency scores for the socio-economic risk mitigation and circular economy pathways suggest that these are critical areas for improvement, highlighting a systemic under-prioritization of social equity and industrial ecology principles within the core decarbonization agenda. At the variable level, the high scores for policy accessibility and incentives indicate a promising move toward transparent and incentive-based governance, although the persistently low scores for institutional coordination present a formidable challenge. The results therefore highlight the urgent need for more balanced institutional involvement, particularly from sector-specialized bodies like the MIIT, to ensure effective policy implementation and address the unique technical and social challenges of hard-to-abate industries.

## Discussion and implications

This section provides a detailed discussion of the core findings from our systematic analysis of China’s industrial decarbonization policies. The discussion is organized into two primary segments to ensure a clear and logical flow. The first segment delves into the empirical discovery of varying policy consistency across different decarbonization pathways, exploring the underlying reasons and theoretical implications of this asymmetry. The second segment builds upon these findings to assess the broader comparative value and structural challenges of China’s policy framework, connecting our specific results to wider debates in decarbonization governance.

### Asymmetric policy consistency across decarbonization pathways

Our bibliometric mapping identifies five distinct policy pathways through which China operationalizes its national industrial decarbonization strategy: energy efficiency, carbon emission abatement, circular economy, scientific and technological innovation, and socio-economic risk mitigation. This multi-pathway architecture reflects a recognition of the transition’s complexity, moving beyond a one-dimensional approach [55]. The framework translates high-level ambitions, such as the 2060 carbon neutrality goal, into focused action streams to address different facets of the challenge [17]. Consequently, our individual evaluation of these pathways using the PMC-Index model provides a more nuanced understanding than a single, aggregate evaluation would allow. This granular view is crucial because it reveals the specific governance logics applied to different transition tasks, a perspective that scholars argue is essential for accurate policy analysis [20].

The most striking finding to emerge from this pathway-specific evaluation is the significant disparity in policy consistency. We identify a clear pattern of asymmetric consistency, indicating that policy consistency and design quality vary significantly across the pathways. Quantitatively, the carbon emission abatement pathway stands apart with a perfect consistency score of 9.07. In contrast, the circular economy and socio-economic risk mitigation pathways trail considerably, with scores of 6.77 and 6.97, respectively, which places them only in the “acceptable” range. This divergence is not random but rather reflects China’s strategic governance priorities. The strong performance of the carbon emission abatement pathway stems from country’s effective use of a hybrid regulatory-market approach [20]. This model powerfully fuses unambiguous top-down mandates, such as sector-specific carbon intensity caps, with sophisticated market mechanisms like the national emissions trading scheme [3, 56, 57]. Furthermore, the efficacy of this pathway is amplified by China’s entrenched hierarchical target responsibility system [3, 17]. Within this system, overarching national objectives are systematically decomposed into binding, quantifiable benchmarks for provincial governments and industrial sectors, thereby creating a robust and cascading chain of accountability that ensures implementation [17, 20].

In a similar vein, the energy efficiency and scientific and technological innovation pathways also demonstrate robust performance, achieving good consistency scores of 8.14 and 8.12. These results indicate a strategic and successful pivot toward a more incentive-based style of governance. Policies within these pathways increasingly rely on economic instruments such as targeted tax rebates for green R&D, tiered electricity pricing to penalize inefficiency, and public–private partnerships to de-risk innovation [20, 22, 40]. This evolution in policy instrument selection aligns with a well-established academic consensus, which posits that deep industrial transformation requires a careful balance between regulatory pressure and the creation of positive economic signals to stimulate corporate investment and behavioral change [3, 20, 58].

In contrast, the socio-economic risk mitigation and circular economy pathways show weaker performance. Their lower consistency scores uncover a fundamental structural issue within the governance framework: the presence of deep-seated policy silos [59]. This term refers to policies developed in isolation, creating a disconnect between decarbonization and other critical objectives like social equity and resource efficiency. A clear illustration of this problem can be seen in the realm of just transition. Programs designed to support workers in coal-dependent regions, such as Shanxi, are frequently implemented as reactive, stand-alone measures—like offering retraining only after a mine has been closed. They are seldom treated as a foundational element and proactively integrated into the regional decarbonization plan from the start [15]. Similarly, the circular economy pathway focuses predominantly on downstream waste management, such as recycling industrial solid waste, while offering insufficient policy support for upstream systemic solutions. These solutions include designing products for longer lifespans and, most importantly, fostering industrial symbiosis networks where one facility’s waste outputs become another’s valuable inputs [60]. This empirical pattern provides strong support for the arguments of scholars like García-García et al. [61], who contend that for a transition to be truly sustainable, policies for decarbonization, social justice, and circularity must be co-designed and intrinsically linked from the very beginning [11, 38, 61].

In summary, the observed pattern of asymmetric consistency provides real-world evidence supporting theories of environmental governance and policy mixes [55, 62]. This theoretical framework posits that managing complex socio-technical transitions requires a diverse portfolio of policy instruments, each tailored to address specific challenges [21, 55, 62]. Our findings thus reveal a key characteristic of China’s state-led decarbonization governance model: its exceptional effectiveness in policy domains aligned with clear, top-down priorities that can be managed through quantifiable, cascading targets. Conversely, these findings highlight an opportunity for learning and refinement in domains that hinge on horizontal collaboration, stakeholder engagement, and adaptive policy integration. This distinction reveals the dynamic nature of the governance model and challenges the utility of universal, one-size-fits-all frameworks for policy evaluation [63].

### Comparative advantages and structural limitations

An international comparison of China’s decarbonization framework highlights its distinctive comparative advantages and structural limitations. This comparative perspective offers practical lessons for both Chinese and global policymakers.

A key strength of the Chinese approach is its regulatory hybridity. This governance model deliberately fuses authoritative state capacity with the dynamic efficiency of market mechanisms [20]. A paradigmatic example of this synergy is the Top-Runner Program. This initiative sets mandatory, technology-forcing energy efficiency standards for key industries, but it simultaneously creates a competitive landscape by offering financial rewards and public recognition to the enterprises that surpass these benchmarks by the widest margin. Evidence suggests that this “carrot and stick” approach can accelerate the adoption of efficient technologies in some heavy industries more effectively than frameworks in other jurisdictions that rely solely on regulation or market signals [64].

Despite this strength, the low consistency score for the circular economy pathway reveals a potential structural gap. China’s current policy framework in this area exhibits a clear emphasis on end-of-pipe solutions, focusing primarily on recycling waste after its generation. However, this approach overlooks the need for policies that incentivize circularity at the design stage and foster industrial symbiosis. Such policies are crucial for promoting durable products and system-level resource exchange [38]. The relative underdevelopment of these upstream and systemic aspects creates a performance gap compared to more holistic international benchmarks. By contrast, the European Union’s Circular Economy Act mandates key principles such as the waste hierarchy, which legally prioritizes waste prevention, and extended producer responsibility, embedding them more deeply into its regulatory fabric [65].

To bridge this gap and enhance policy consistency for the circular economy, policy interventions should focus on three key areas. First, deeper legislative reforms are needed to institutionalize extended producer responsibility (EPR) in sectors such as manufacturing and electronics. This would involve coupling binding eco-design mandates with rigorous, verifiable compliance with the waste hierarchy [65]. Second, establishing a transparent and tiered certification system for industrial symbiosis parks, backed by tangible fiscal incentives like significant tax rebates for demonstrated high levels of closed-loop material utilization, would create a powerful market pull for circular practices [60]. Third, to overcome the current fragmentation, consolidating the dispersed oversight of circular economy initiatives under a dedicated inter-ministerial authority with a clear mandate could significantly mitigate the regulatory incoherence that currently hinders systemic progress [66].

Similarly, the socio-economic risk mitigation pathway also requires improvement. Although China has pioneered instruments like regional transition funds for coal-dependent areas, its overall framework is less comprehensive than leading international models. In practice, stakeholder engagement remains largely informal, transition planning is regionally fragmented, and workforce development strategies are not fully synchronized with decarbonization timelines [15]. Addressing these gaps requires learning from mechanisms like the European Union’s Just Transition Mechanism, which prioritizes formal involvement of local communities, social partners, and businesses in planning [67]. Specifically, progress is needed in three areas: first, institutionalizing meaningful multi-stakeholder engagement to improve transition planning; second, developing a nationally coordinated but flexible transition framework to align local economic diversification with national decarbonization goals; and finally, implementing long-term workforce development programs timed to match sector-specific decarbonization roadmaps.

While the comparative analysis and optimization suggestions derived from our PMC-Index evaluation are insightful, it is crucial to acknowledge the limitations of the methodological approach itself. The PMC-Index offers a valuable framework for evaluating the comprehensiveness and internal consistency of decarbonization policies, though it inherently focuses on structural design rather than functional interactions among policy instruments. This methodological approach effectively captures the scope and coherence of individual policy pathways, but it does not address how these policy instruments interact during implementation, including their potential synergistic, neutral, or counteractive effects. This characteristic of the PMC-Index merits consideration in China’s multi-level governance context, where policies often span multiple sectors and administrative levels, creating a complex landscape of potential interactions. Future studies could therefore integrate analytical methods capable of capturing these dynamic interrelationships, such as qualitative comparative analysis or policy network modeling, to reveal how instrument interactions shape decarbonization outcomes. Bridging this gap between policy design and operational interplay would further advance the understanding of complex industrial decarbonization and carbon neutrality governance.

## Conclusions

This study pioneers a comprehensive evaluation of China’s industrial decarbonization policy framework by innovatively synthesizing bibliometric analysis with the Policy Modeling Consistency (PMC) Index. Our pathway-specific consistency assessment directly responds to major scholarly critiques of universal policy metrics. This methodology, in contrast to conventional aggregate-level analyses, offers a more nuanced approach to policy evaluation.

The findings reveal a diversified policy landscape with varying degrees of consistency across different decarbonization pathways. While the carbon emission abatement pathway shows perfect consistency (9.07), reflecting China’s strategic prioritization of direct emission control measures, other pathways such as energy efficiency (8.14) and scientific and technological innovation (8.12) demonstrate good consistency. However, the socio-economic risk mitigation (6.97) and circular economy (6.77) pathways exhibit only acceptable consistency, indicating relative weaknesses in integrating social equity and industrial ecology principles. Based on these findings, we propose two targeted policy interventions to enhance governance effectiveness: (1) Institutionalize just transition mechanisms via regionally differentiated retraining programs and adaptive social safety nets; (2) Strengthen circular economy integration through sector-specific material flow regulations and cross-industrial symbiosis networks.

The research carries significant implications for both policymakers and scholars. From a practical perspective, the findings highlight the necessity of pathway-specific interventions to address policy inconsistencies while maintaining industrial competitiveness and social equity. Theoretically, our findings advance environmental governance frameworks by demonstrating the critical role of policy consistency in industrial decarbonization.

## Supplementary Information

Additional file 1

## Data Availability

The author confirms that all data generated or analysed during this study are included in the Additional file 1 of this article.
