# Supplementary material for: Navigating carbon neutrality: policy pathways and consistency on industrial decarbonization in China
Source: Carbon Balance Manag. 2025 Dec 14;20:66. doi: 10.1186/s13021-025-00356-7 (PMC12739856; doi:10.1186/s13021-025-00356-7)
Supplement: Supplementary file 1 — Additional file 1 [file 13021_2025_356_MOESM1_ESM.docx]

Supplementary Information: Policy data.

| No. | Policies | Formulators |
| --- | --- | --- |
| 1 | Accelerating the Establishment of a Unified and Standardised Carbon Emission Statistics and Accounting System | National Development and Reform Commission, et al. |
| 2 | Action Plan for Carbon Peaking by 2030 | State Council of China |
| 3 | Action Plan for Green and Low Carbon Development of the Information and Communication Sector | Ministry of Industry and Information Technology, et al. |
| 4 | Action Plan for Technology and Innovation for Carbon Neutrality in Higher Education | Ministry of Education |
| 5 | Benchmarking Levels in Key Areas of Clean and Efficient Utilisation of Coal | National Development and Reform Commission, et al. |
| 6 | Carbon Emissions Trading Management Measures (Trial) | Ministry of Ecology and Environment |
| 7 | China’s Policies and Actions to Address Climate Change | State Council of China |
| 8 | Guidance on Accelerating the Establishment of the Sound Green Low-Carbon Cycle Development of the Economic System | State Council of China |
| 9 | Guidance on Coordinating and Strengthening the Work Related to Climate Change and Ecological Protection | Ministry of Ecology and Environment |
| 10 | Guidelines for the Construction of Standard System for Carbon Peaking and Carbon Neutrality | National Standardization Administration et al. |
| 11 | Guidelines for Verification of Greenhouse Gas Emission Reports by Enterprises (Trial) | Ministry of Ecology and Environment |
| 12 | Guiding Opinions on Promoting High-Quality Development of Central Enterprises in Carbon Peaking and Carbon Neutrality | State-owned Assets Supervision and Administration Commission of State Council |
| 13 | Guiding Opinions on Strengthening Classification and Guiding the Cultivation of New Initiatives for the Transformation and Development of Resource-Based Cities | National Development and Reform Commission |
| 14 | Implementation programme for building a national education system for green and low-carbon development | Ministry of Education |
| 15 | Implementation Programme for Carbon Peaking and Carbon Neutrality under the Support of Science and Technology | Ministry of Science and Technology, et al. |
| 16 | Implementation Programme for Carbon Peaking in Building Materials Sector | Ministry of Industry and Information Technology, et al. |
| 17 | Implementation Programme for Carbon Peaking in Industry Sectors | Ministry of Industry and Information Technology, et al. |
| 18 | Implementation Programme for Carbon Peaking in Non-Ferrous Metals Sector | Ministry of Industry and Information Technology, et al. |
| 19 | Implementation Programme for Carbon Peaking in Urban and Rural Construction Sectors | Ministry of Housing and Urban-Rural Development, National Development and Reform Commission |
| 20 | Implementation Programme for Promoting Green and High-Quality Development of Data Centres, 5G and Other New Infrastructure under the Carbon Peaking and Carbon Neutrality Goals | National Development and Reform Commission, et al. |
| 21 | Implementation Programme for the Establishment of a Sound Standardized Measurement System for Carbon Peaking and Carbon Neutrality | State Administration for Market Regulation, et al. |
| 22 | Implementation Programme for the Transformation and Upgrading of Coal Power Units | National Development and Reform Commission, National Energy Administration |
| 23 | Implementation Programme on Emission Reduction and Carbon Sequestration in Agriculture and Rural Areas | Ministry of Agriculture and Rural Affairs, National Development and Reform Commission |
| 24 | Implementation Programme on Promoting High-Quality Development of New Energy in the New Era | National Development and Reform Commission, National Energy Administration |
| 25 | Implementation Programme on Synergies between Pollution Reduction and Carbon Reduction | Ministry of Ecology and Environment, et al. |
| 26 | Institutional Programme to Control Energy Consumption Intensity and Quantity | National Development and Reform Commission |
| 27 | Integrating Carbon Peaking and Carbon Neutrality into the Overall Layout of Ecological Civilization Construction | Ministry of Ecology and Environment |
| 28 | Interim Provisions on the Administration of Carbon Emission Trading | State Council of China |
| 29 | National Action Programme on Health Adaptation to Climate Change | National Bureau of Disease Control and Prevention |
| 30 | National Programme for Carbon Peaking Pilot Construction | National Development and Reform Commission |
| 31 | Notice on Accelerating the Comprehensive Utilisation and Demonstration of Bulk Solid Waste | National Development and Reform Commission |
| 32 | Notice on Actively Assisting Employees in Employment and Entrepreneurship in Resolving Overcapacity | State Administration for Industry and Commerce |
| 33 | Notice on Full Coverage of Renewable Energy Green Power Certificates and the Promotion of Renewable Energy Power Consumption | National Development and Reform Commission, et al. |
| 34 | Notice on Further Strengthening the Implementation of Updating and Application of Energy Saving Standards | National Development and Reform Commission, State Administration for Market Regulation |
| 35 | Notice on Promoting the Work Related to Carbon Peaking and Carbon Neutrality in National Eco-Industrial Demonstration Parks | Ministry of Ecology and Environment |
| 36 | Opinions on Accelerating the Comprehensive Green Transformation of Economic and Social Development | Central Committee of the Communist Party of China, State Council of China |
| 37 | Opinions on Comprehensively Promoting the Construction of a Beautiful China | Central Committee of the Communist Party of China, State Council of China |
| 38 | Opinions on Financial Support for Carbon Peaking and Carbon Neutrality | Ministry of Finance |
| 39 | Opinions on Promoting Supply-Side Structural Reform and Preventing and Resolving the Risks of Overcapacity in Coal and Power Generation | National Development and Reform Commission, et al. |
| 40 | Opinions on Promoting the Green Development of Urban and Rural Construction | Central Committee of the Communist Party of China, State Council of China |
| 41 | Opinions on the Complete and Accurate Implementation of the New Development Concept and Carbon Peaking & Carbon Neutrality | Central Committee of the Communist Party of China, State Council of China |
| 42 | Opinions on the Resettlement of Employees in the Process of Resolving Overcapacity in the Iron and Steel and Coal Industries and Realising Development Out of Difficulties | Ministry of Human Resources and Social Security, et al. |
| 43 | Regulations on Ecological Protection Compensation | State Council of China |
| 44 | Report of the 20th National Congress of the Communist Party of China (excerpt) | Central Committee of the Communist Party of China |
| 45 | Special Action Plan for Energy Saving and Carbon Reduction in Iron and Steel Industry | National Development and Reform Commission, et al. |
| 46 | Special Action Plan for Energy Saving and Carbon Reduction in Aluminium Electrolysis Industry | National Development and Reform Commission, et al. |
| 47 | Special Action Plan for Energy Conservation and Carbon Reduction in Oil Refining Industry | National Development and Reform Commission, et al. |
| 48 | Special Action Plan for Energy Conservation and Carbon Reduction in Ammonia Industry | National Development and Reform Commission, et al. |
| 49 | Special Action Plan for Energy Saving and Carbon Reduction in Cement Industry | National Development and Reform Commission, et al. |
| 50 | The 14th Five-Year Plan for Circular Economic Development | National Development and Reform Commission |
| 51 | The 14th Five-Year Plan for East-West Cooperation in Science and Technology | Ministry of Science and Technology, et al. |
| 52 | The 14th Five-Year Plan for Energy Conservation and Emission Reduction | State Council of China |
| 53 | The 14th Five-Year Plan for Modern Energy System | National Development and Reform Commission, National Energy Administration |
| 54 | The 14th Five-Year Plan for National Cleaner Production Promotion Programme | National Development and Reform Commission, et al. |
| 55 | The 14th Five-Year Plan for the Green Development of Industry | Ministry of Industry and Information Technology, et al. |
| 56 | The 14th Five-Year Plan for the National Economic and Social Development of China and the Outline of Long-Term Goals for 2035 (Excerpt: Actively Responding to Climate Change) | Central Committee of the Communist Party of China |
| 57 | Work Programme for Accelerating the Construction of a Dual Control System for Carbon Emissions | State Council of China |
| 58 | Work Programme on Strengthening the Construction of Carbon Peaking and Carbon Neutrality in Higher Education Talent Cultivation System | Ministry of Education |

Source: Collected and presented by the authors.
